# Proteomic Approaches to Unravel the Molecular Dynamics of Early Pregnancy in Farm Animals: An In-Depth Review

**DOI:** 10.3390/jdb12010002

**Published:** 2023-12-30

**Authors:** Shradha Jamwal, Manoj Kumar Jena, Nikunj Tyagi, Sudhakar Kancharla, Prachetha Kolli, Gowtham Mandadapu, Sudarshan Kumar, Ashok Kumar Mohanty

**Affiliations:** 1 Proteomics and Structural Biology Lab, Animal Biotechnology Centre, National Dairy Research Institute, Karnal 132001, Haryana, India; ishuangel17@gmail.com (S.J.); tyaginikunj458@gmail.com (N.T.); kumarsudershan@gmail.com (S.K.); 2 Department of Biotechnology, School of Bioengineering and Biosciences, Lovely Professional University, Phagwara 144411, Punjab, India; drmanoj.jena@gmail.com; 3 Devansh Lab Werks, 234 Aquarius Drive, Homewood, AL 35209, USA; sudhakar@devlabwerks.com (S.K.); gowtham@devlabwerks.com (G.M.); 4 Microgen Health Inc., 14225 Sullyfield Cir Suite E, Chantilly, VA 20151, USA; prachetha@microgenhealth.com; 5 ICAR–Central Institute for Research on Cattle, Meerut Cantt 250001, Uttar Pradesh, India

**Keywords:** early pregnancy, proteomics, mass spectrometry, embryo implantation, farm animals

## Abstract

Infertility is a major problem in farm animals, which has a negative economic effect on farm industries. Infertility can be defined as the inability of animals to achieve a successful pregnancy. Early pregnancy is crucial to establish a successful pregnancy, and it is reported that 70–80% and 20–30% of total embryonic loss occur in cattle and pigs, respectively, during the first month of pregnancy. The advanced high-throughput proteomics techniques provide valuable tools for in-depth understanding of the implantation process in farm animals. In the present review, our goal was to compile, assess, and integrate the latest proteomic research on farm animals, specifically focused on female reproduction, which involves endometrial tissues, uterine fluids, oviductal fluids, and microRNAs. The series of studies has provided in-depth insights into the events of the implantation process by unfolding the molecular landscape of the uterine tract. The discussed data are related to pregnant vs. non-pregnant animals, pregnancy vs. oestrous cycle, different days of the early pregnancy phase, and animals with uterine infections affecting reproduction health. Some of the studies have utilized non-invasive methods and in vitro models to decipher the molecular events of embryo-maternal interaction. The proteomics data are valuable sources for discovering biomarkers for infertility in ruminants and new regulatory pathways governing embryo-uterine interaction, endometrium receptivity, and embryonic development. Here, we envisage that the identified protein signatures can serve as potential therapeutic targets and biomarkers to develop new therapeutics against pregnancy diseases.

## 1. Introduction

**Figure 1:**
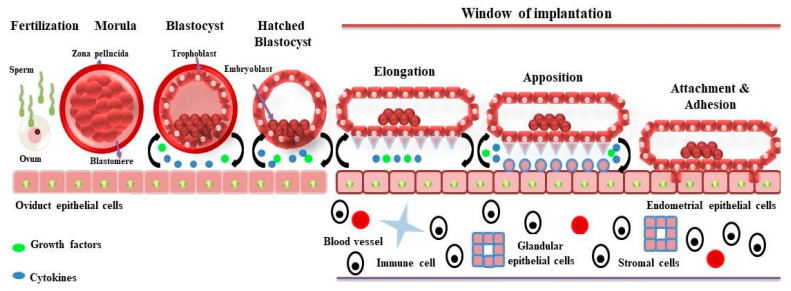
The process of implantation into the maternal endometrium in domestic ruminants occurs in various steps that involve fertilization, blastocyst hatching, elongation, apposition, attachment, and adhesion. During these phases, the conceptus and/or endometrium release several growth factors and cytokines, including interferon tau, into the uterine microenvironment. These molecules act in an autocrine and paracrine manner so that proper communication can occur between the conceptus and the uterine endometrium for the establishment of a successful pregnancy.

Implantation is a highly coordinated and intricately timed mechanism that requires an essential cross-signaling between blastocyst and maternal endometrium to establish a successful pregnancy in mammals, including domestic animals (cattle, sheep, pigs, and goats), humans, and primates [1]. The process of implantation varies among species, and it has been divided into three categories based on the series of events taking place during interaction between blastocyst and uterine cells. The three categories are centric, eccentric, and interstitial types of implantation [2]. The centric type of implantation occurs in domestic animals, where blastocysts grow in size and fuse with the apical membrane of luminal epithelia without invading it. On the other hand, in eccentric and interstitial types of implantations, blastocysts invade the luminal epithelia partially and fully, respectively [3]. The eccentric type of implantation occurs in rodents, including rats, mice, and hamsters, whereas humans and guinea pigs involve an interstitial type of implantation [2]. The general mechanism of implantation (Figure 1) is conserved across mammals and follows the shedding of the zona pellucida, orientation, apposition, attachment, and adhesion of the blastocyst to the endometrium; however, after fertilization, the timing of the blastocyst’s development and subsequent series of implantation stages vary among species, most importantly the window of implantation (WOI), where the uterus allows the blastocyst to attach and fuse [3,4]. In cattle, blastocysts are formed after 7–8 days post-fertilization and do not implant until 30 days post-fertilization. In pigs, blastocysts are formed after 6 days post-fertilization, and implantation takes place 20–22 days post-fertilization [5]. In sheep, blastocysts are formed after 6 days post-fertilization, and implantation takes place after 22 days post-fertilization [3].

Infertility is a major problem in farm animals, which has a negative economic effect on farm industries. It can be defined as the inability of animals to achieve a successful pregnancy. Various factors are implicated in infertility, such as nutrition, genetics, environmental factors, and maternal factors like incompetent embryo, loss of embryo, lack of signals during cross-talk between embryo and uterine endometrium, and non-receptive state of uterine endometrium [6]. The peri-implantation period is considered critical for embryo development and endometrium receptivity in ruminants, as most of the pregnancy losses occur in this period [7]. In cattle, early embryonic mortality is explained as the death of fertilized ova and embryos up to day 28 of gestation [8]. After a successful insemination, 70–80% of total embryonic loss occurs in cattle during the first three weeks of post-insemination [9], whereas in pigs, the prenatal death rate accounts for 30–40% on average, with the largest loss of 20–30% during the first month of gestation [10]. It is necessary to improve the reproductive efficiency of female animals to reduce the economic loss in farm industries. Thus, an in-depth understanding of molecular mechanisms and signaling pathways involved in the processes of post-hatching development, endometrial receptivity, and implantation is essential to develop therapeutic strategies for successful pregnancy in livestock.

“Omics”-based high-throughput techniques have emerged as promising tools to understand the underlying mechanisms of a biological system, which provide information on the global contents of proteins, lipids, RNAs, and metabolites in cells or tissues [11]. Based on the study of cellular contents, “omics”-based studies have been categorized as follows: Proteomics (global profiling of proteins), transcriptomics (global profiling of RNA transcripts), metabolomics (global profiling of metabolites), and lipidomics (global profiling of lipids) [12] Proteins are the primary functional biomolecules of cells [13], and the expression and state of proteins give rise to different physiological processes at the cellular level [14]. The term ‘proteome’ is defined as the total protein content present in a cell or an organism throughout their entire lifespan [15,16]. A single gene encodes a single protein or variants of proteins; however, a single protein exhibits different functions depending on expression, cellular localization, and post-translational modification (PTM) [16].

**Figure 2:**
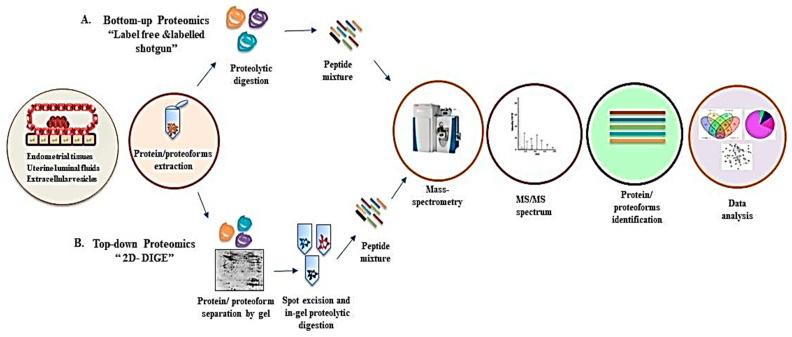
A generalized overview of mass spectrometry-based proteomics analysis of different sources from the early phase of pregnancy shows various steps involved in proteome analysis by two approaches, which are as follows: (**A**) Bottom-up proteomics: protein extraction, enzymatic digestion of protein/proteoforms followed by mass spectrometry analysis, identification of proteins, and data analysis using different bioinformatics tools. (**B**) Top-down proteomics: protein/proteoform extraction, gel separation, spot excision, proteolytic digestion followed by identification by mass spectrometry, and data analysis.

The term ‘proteoform’ is being used to define the complexity of ‘proteome’ due to variation in single proteins [17]. Thus, a ‘proteome’ can be defined as a set of total proteoforms expressed in biological material (specific tissue, fluids, cell type, or organelle) at a particular time point [16]. The proteomic approaches provide systematic analysis of proteoforms, which constitute a parts of the proteome, as well as quantitative analysis of protein abundance [18,19]. The mass-spectrometry (MS)-based approaches, namely top-down and bottom-up proteomics, are used for proteomic analysis [18,20]. The top-down approach is demonstrated by 2-D DIGE (2-dimensional Difference Gel Electrophoresis), and MS/MS techniques involve separation of intact proteins and their proteoforms on the basis of their pI (isoelectric point) and MW (molecular weight), followed by excision of protein gel, proteolytic digestion, and MS analysis [18]. The bottom-up proteomics is a more widely used technique that involves proteolytic digestion of proteins into small peptides, separation by liquid chromatography, ionization, and MS analysis [20]. Proteomics-based technologies have been adopted to understand the early phase of pregnancy in farm animals. A generalized overview of the workflow for top-down and bottom-up proteomics for proteomic analysis of early pregnancy is shown in Figure 2. The objective of this review is to summarize the current data from proteomics-based studies on mechanisms involved in embryo implantation and endometrium receptivity in various farm animals (bovine, sheep, and pigs). A total of 614 articles were identified when searched on Google Scholar and PubMed and published before 1st January, 2023. The terms used for the search were “proteomics”, “early pregnancy”, “endometrium”, “bovine”, “pig”, and “sheep”. The inclusion criteria included studies discussing the early phase of pregnancy and sources for proteomics studies such as endometrial tissue, oviductal fluid, and uterine luminal fluids relating to implantation/uterine receptivity and uterine infections in farm animals (bovine, sheep, and pig). The exclusion criteria involved human studies, follicular fluids, blastocyst, and male reproduction studies. After screening the searched articles, a total of 54 research articles (Bovine = 34, sheep = 13, and Pig = 7) were selected for this review article. In addition, 95 articles were included in this manuscript, corresponding to the discussions and analysis of the data.

## 2. Proteomics of Bovine Uterus Milieu to Decipher the Molecular Landscape of Successful Pregnancy

Receptive endometrium is vital for appropriate fetal-maternal communication to establish a successful pregnancy. Bovine endometrium, based on its structure and function, is divided into two layers, namely the basal layer and the functional layer with pseudostratified columnar/simple columnar epithelial cells [21]. The endometrium undergoes morphological and functional changes throughout the estrous cycle to receive a viable competent blastocyst [22] and remains under the influence of ovarian hormones (estradiol-17β and progesterone). Progesterone plays a key role in the maintenance of pregnancy [23]. The endometrium is transformed from a non-receptive to a receptive state when it comes in contact with floating blastocysts and is known to be affected by various signaling molecules released from blastocysts and endometrial cells [24]. To this date, it is unclear how embryo implantation is regulated in ruminants [25]. Therefore, it is necessary to identify molecules and signaling pathways associated with endometrial receptivity and embryo implantation. This may lead to the discovery of molecular markers as predictors of implantation and pregnancy, which would further pave the way for finding therapeutic solutions to treat reproductive disorders. Several studies have attempted to investigate the molecular signature associated with uterine endometrium using proteomics techniques during fetal-endome trium interaction and at different time points of pregnancy (Table 1). Proteome profiling and quantitative proteomics have been carried out on endometrial tissue, uterine flushing, oviductal fluid (OF), and blood during different stages of pregnancy in bovines. A review of the proteome of bovine endometrium, oocytes, and early embryos by Deutsch et al. has presented substantial information on proteins involved in physiological processes [26].

### 2.1. Proteome Profiling of Endometrial Tissue

A study on cow endometrial tissue from pregnant and non-pregnant animals at the onset of the pre-attachment period, specifically on the 18th day of pregnancy, using quantitative proteomics-based approaches (2-D DIGE) and matrix-assisted laser desorption ionization-time-of-flight/time-of-flight mass spectrometry (MALDI-TOF/TOF MS) revealed interesting findings [36]. Four proteins, namely Rho GDP dissociation inhibitor beta (Rho GDI), 20 alpha-hydroxysteroid dehydrogenase (20 alpha HSD), isocitrate dehydrogenase (ICD), and acyl-CoA binding protein (ACBP), were identified with a two-fold increased abundance in pregnant animals in comparison to non-pregnant animals [36]. The abundance of these proteins suggests that they play an important role during the implantation process in ruminants. Isocitrate dehydrogenase (ICD) is an enzyme of the Krebs cycle, and Rho GDI is a member of the Rho guanosine diphosphate dissociation inhibitors family, which inhibits the GDP/GTP exchange [49]. Acyl-CoA binding protein (ACBP), also known as diazepam binding inhibitor, is an acyl-CoA binding protein that plays an essential role in lipid metabolism. The absence of the ACBP gene induces embryonic lethality during the early pre-implantation stage [50]. The enzyme 20-Alpha-HSD is involved in prostaglandin catabolism in bovine endometrium [36]. It is suggested in the mice study that maternal and fetal 20-Alpha-HSD are essential for the maintenance of pregnancy. The deletion of enzyme 20-Alpha-HSD increased the progesterone level, which is lethal for fetus survival [51].

The impact of embryonic mortality varies among species; for instance, early embryonic mortality is more prevalent in cattle, which occurs before Day 19 of pregnancy, whereas it has limited effects on buffaloes [52]. Late embryonic mortality (from Day 25 to Day 32) is a major cause of poor reproductive efficiency in buffaloes [52,53]. Till 2013, the proteome of the buffalo embryo-uterine interaction or uterine environment was unknown to our knowledge. For the first time, Balestrieri et al. employed 2D-DIGE and MALDI-ToF mass spectrometry techniques to elucidate the proteomic profiles of the chorioamnions and uterine caruncles in the water buffalo [35]. They compared the difference in protein expression of the chorioamnion and the corresponding caruncles of retarded embryos to those of normal embryos (on day 27 of gestation) [35]. A total of 95 differentially expressed protein spots (*p* < 0.05) were identified in tissue from normal and retarded embryos by 2D-DIGE analysis, and 93 differentially expressed protein spots (*p* < 0.05) in normal and retarded car uncles [35]. Further analysis by MALDI-ToF mass spectrometry revealed a total of 18 proteins (normal and retarded embryos) and 17 proteins (normal caruncles and retarded caruncles) with a significant total ion score confidence interval. Gene ontology (GO) of differentially expressed proteins (DEPs) in terms of molecular functions in the chorioamnions revealed their association with different molecular functions such as DNA and RNA binding, cell redox homeostasis, protein folding, cytoskeletal organization, proteolysis regulation, chromosome segregation, protein transport, and calcium binding. The molecular function analysis of identified proteins in caruncles showed their involvement in protein folding, hemoglobin binding, proteolysis regulation, and the nucleoside metabolic process [35].

### 2.2. Proteome Profiling of Uterine Lumen Fluid (ULF)

Endometrial tissue is widely used in the study of reproductive biology; however, obtaining an endometrial biopsy could have a detrimental effect on embryo implantation. There fore, less invasive methods such as uterine fluids can be a great alternative to exploring the process of implantation and other reproduction mechanisms [54]. In addition, there is a possibility that the results obtained from the biopsy will not match the subsequent cycles of pregnancy [55]. Uterine lumen fluid (ULF) is highly dynamic and contains various components such as hormones, enzymes, cytokines, growth factors, protein carriers, amino acids, and other substances collectively known as histotroph. These substances are synthesized and secreted by uterine cells, namely epithelial cells (luminal and glandular cells) and stromal cells [56]. These secretions are crucial for the establishment and maintenance of pregnancy in mammals. In a recent report, Simintiras et al. used high-throughput untargeted semi-quantitative metabolomic profiling on ULF and showed that uterine fluid is metabolically semi-autonomous and has a role in the active metabolic pathways due to the presence of active enzymes [57]. Their study found 317 and 7 more metabolites on days 12 and 16, respectively, in ULF from cyclic heifers through metabolomic profiling, and these metabolites were associated with fertility and pregnancy [57]. Various methodologies from different studies have been summarized and reviewed in detail by Itze-Mayrhofer and Brem, revealing the processes for sample preparation and the workflow of proteomics analysis of uterine fluids (UF) in different farm animals [58].

#### 2.2.1. Proteomics of Uterine Lumen Fluid (ULF) in In Vivo Studies

The first study using ULF was reported by Ledgard et al., who compared the ULF proteome of pregnant cows with that of nonpregnant cows during the peri-attachment period (specifically on days 16 and 18) [27]. By using the 2D-DIGE approach, 9 DEPs with high abundance were identified in pregnant cows, whereas 4 proteins had low abundance. The abundant proteins were mainly involved in biosynthetic pathways, cytoskeleton organization, the Kreb cycle, and antioxidant activity. The less abundant proteins were found to be associated with endometrial remodeling. The proteins obtained in endometrial biopsy proteome analysis by Berendt et al. showed the least similarity with this study, and only one common protein, isocitrate dehydrogenase (ICD), was observed [36]. A study on the effect of the uterine environment at different stages of the oestrous cycle on blastocyst development post-hatch using the 2D-DIGE approach identified 10 DEPs on ULF between Day 5 and Day 9 after oestrus [59].

With advancements in proteomics-based approaches, Faulkner et al. employed isobaric tags for relative and absolute quantitation (iTRAQ)-based quantitative proteomics to characterize bovine endometrium [30]. In their study, they compared the UF with blood plasma from beef heifers on day 7 of the oestrous cycle. In total, 112 proteins were identified in UF and plasma, out of which 53 (in UF, 35 proteins upregulated and 18 proteins downregulated) DEPs (minimum fold change of ±1.5 or greater and FDR < 0.10) were selected for analysis. The identified DEPs were mostly metabolic enzymes, anti-oxidants, and immune response modulators, which were associated with 12 different biological processes, demonstrating the multifunctional role of the uterine proteome [30]. Using the same quantitative proteomics-based approach, Faulkner et al. investigated the effects of progesterone and stage of the oestrous cycle on the uterine proteome in beef heifers on day 5 of the oestrous cycle [31]. For proteome profiling, the UF were collected from animals at days 7 and 15 of the oestrous cycle and divided into low and high progesterone groups. A total of 169 proteins were identified on each day of the cycle, out of which 40 were DEPs identified on Day 15 compared to Day 7. There were 20 up-regulated proteins (5.9-fold higher, *p* < 0.05, FDR < 0.10) and 20 down-regulated proteins (2.3-fold lower, *p* < 0.05, FDR < 0.10) present in the uterine proteome on Day 15, which were associated with different biological processes including apoptosis, cell communication, cell cycle, cellular processes, developmental process, immune system, cellular transport, homeostatic process, response to stimuli, and metabolic process [31]. It was observed that progesterone has an effect on protein expression at different days of the cycle (days 3–7 or 15) [31]. Similarly, the study by Mullen et al., using a gel-free approach to globally characterize the bovine uterine proteome at different stages of the oestrous cycle, revealed 300 proteins (on Day 7) and 510 proteins (on Day 13), identified in high-fertility cattle by utilizing a label-free liquid chromatography−tandem mass spectrometry (LC−MS/MS) approach [29]. The bioinformatics analysis showed that proteins from both groups were majorly involved in metabolism, multicellular development, transport, and signal transduction [29]. This study revealed two novel proteins in UF on Day 13, namely S100-A8 and S100-A9, which belong to the S100 family of molecules, are inflammatory proteins with affinity to Ca2+ ions [60], and play an important role at the maternal-fetal interface. The altered expression of these proteins is known to be lethal for pregnancy establishment [61]. These above-mentioned studies [29,30,31] described the proteome profile of uterine fluid in pregnant cows at a single or two points on days 5, 7, 13, 16, 15, and 18. A study on the global protein content in UF from days 10, 13, 16, and 19 by using iTRAQ-based quantitative analysis revealed proteins such as RPB4 (DNA-dependent RNA polymerase), TIMP2 (tissue inhibitor of metalloprotease-2), IFNT (interferon Tau), ALDOA (aldolase A), cytochrome C oxidase, GSN (gelsolin), HSP90A1 (heat shock protein 90-alpha), SERPINA31 (alpha-1 antitrypsin), PNP (purine nucleoside phosphorylase), and HSPA8 (heat shock protein family A (Hsp70) member 8) proteins present during the pre-implantation period of pregnancy in cows [32].

In another attempt to explore the protein profile of UF in relation to embryo growth and development, Beltmen et al. compared the UF of beef heifers with that of normal and degenerate embryos on Day 7 after insemination using LC-MS technology [62]. Out of 40 identified proteins, six up-regulated proteins were found in viable embryos, and one up-regulated protein was found in degenerate embryos [62]. Unique proteins such as platelet-activating factor acetyl hydrolase 1b catalytic subunit 3 (PAFAH1B3) were found in the animal group with viable embryos. The protein PAFAH1B3 is a subunit of platelet-activating factor acetyl hydrolase (PAF-AH), which plays crucial roles in various physiological processes such as apoptosis, wound healing, fertilization, implantation, and embryonic development [63,64,65]. S100-A4, the only highly abundant protein found in groups with degenerate embryos, is also known as metastasin 1 (Mts1) or fibroblast-specific protein 1 (Fsp1) and is involved in a variety of biological functions, such as cell motility, adhesion, and invasion [66]. This protein is also known to be involved in the progression and metastasis of cancer [67]. It is not clear how the S100-A4 protein regulates embryonic development and endometrial function during the onset of implantation, which needs further study. In another study, Fortes et al. employed RNA-sequencing on uterine tissue (for transcriptome profiling) and MS for proteome profiling of UF to relate the protein and gene expression patterns of the uterus and its secretion during puberty and unravel the molecular dynamics during implantation and pregnancy [68]. This study made a comparison of the proteome and transcriptome data of pre-pubertal and post-pubertal cycling *Bos indicus* heifers and found 4 DEPs encoded by differentially expressed genes in post-pubertal heifers: OVGP1 (oviduct-specific glycoprotein), GRP (gastrin-releasing peptide), CAP1 (cyclase-associated actin cytoskeleton regulatory protein 1), and HBA (hemoglobin alpha 2). It is suggested that these proteins may help the uterus prepare for a successful pregnancy. OVGP1 (oviduct-specific glycoprotein 1) is an estrogen-dependent protein that is found in the oviductal fluid of different mammalian species, including bovine. OVGP1 (oviduct-specific glycoprotein 1) plays an important role in the processes of fertilization, sperm capacitation, and embryonic development [69,70]. During fertilization, OVGP1 binds to the zona pellucida of oocytes and modifies the matrix structure to allow sperm penetration [71]. In a study with a human endometrial epithelial cell line, it was found that OVGP1 regulates the expression of receptivity-related genes, including cytokines, matrix metalloproteases, and tissue inhibitors of matrix metalloproteases [72]. CAPs (cyclase-associated actin cytoskeleton regulatory protein) are an actin-binding protein that, together with ADF/cofilin, regulates actin dynamics [73]. Cofilin, also an actin-binding protein (a component of actin cytoskeleton proteins), plays an important role in endometrium remodeling during blastocyst implantation [46]. The protein GRP is released by uterine glandular cells into the uterine lumen and regulates pregnancy through autocrine and paracrine modes of signaling [74]. Moraes et al. compared the UF from high fertility to sub-fertile or infertile heifers on Day 17 [75]. In total, 221 DEPs were found in pregnant high-fertile and sub-fertile heifers, out of which 142 were up-regulated and 79 were down-regulated [75]. These DEPs were associated with vitamin B6 metabolism, energy metabolism, the p38 MAPK pathway, cytoskeletal regulation, hemostasis, the plasminogen activating cascade, and blood coagulation pathways [75]. The differential expression of proteins among these two groups indicated that these proteins alter the uterine environment and influence the uterine receptivity for successful implantation and the survival of the fetus.

To enhance the milk yield in dairy cows, single trait selection has been extensively used; however, it has led to a decline in fertility in high-yielding dairy cows [76,77]. In a recent study, Gegenfurtner et al. investigated the effect of genetic merit for fertility on the proteome of the bovine uterine luminal fluid from Day 19 of pregnancy [77]. Using nanoLC-MS/MS coupled with a label-free quantification approach, they identified 597 DEPs between the three groups: heifers with a low fertility index (Holstein) and two groups of heifers with a high fertility index (Holstein and Montbéliarde) [77]. Sorbitol dehydrogenase (SORD) was highly abundant (with a 14.3- and 11.1-fold expression) in high fertility groups (Holstein and Montbéliarde) in comparison to low fertility groups (Holstein) [77]. The protein SORD (sorbitol dehydrogenase) is required for fructose synthesis from glucose. Fructose is the most abundant hexose sugar in the conceptus and endometrium of porcines and may play an important role in the growth and development of the fetus [78]. SORD (sorbitol dehydrogenase) is expressed by porcine uterine epithelial cells during the peri-implantation stage, indicating its role in implantation [77].

The postpartum and peripartum periods are critical stages for reproductive health and are decisive factors for fertility [79]. Aranciaga et al. explored the ULF from early to mid-postpartum (first to third estrus) to analyze the effects of these molecular changes on embryonic development [33]. LC–MS/MS (Liquid chromatography with tandem mass spectrometry) analysis identified a total of 1563 proteins, of which 472 were novel in bovine ULF at day 7 of pregnancy at the first and third estrus postpartum. Gene ontology (GO) revealed these proteins to be involved in metabolic processes, regulatory pathways, responses to stress, and immunomodulation [33]. The proteins that were identified in ULF and have an impact on embryo quality are eukaryotic translation initiation factor 5A1, plasminogen, membrane-associated guanylate kinase inverted 3, phospholipase A2 activating protein (PLAA), triosephosphate isomerase (TIM), delta-amino levulinic acid dehydratase (ALADH), costars family protein (ABRACL) dihydropteridine reductase, cystatin B, acyl-CoA-binding protein (DBI), protein S100A2, Unc-80 homolog and NALCN channel complex subunit, ATP synthase subunit beta, macrophage migration inhibitory factor (MIF), pyruvate kinase (PKM2), alpha-1-antiproteinase (SERPINA1), prostaglandin reductase 1 (PTGR1), and myostatin (MSTN) [33].

#### 2.2.2. Proteomics of Uterine Lumen Fluid (ULF) in In Vitro Studies

In vitro models utilizing the co-culture of endometrial cells and embryos or trophoblast cells provide a great opportunity to study the implantation process at different time intervals, which is difficult to investigate in in vivo studies. Moreover, the in vitro models are easy to manipulate and readily available [80]. Several powerful techniques have been developed over the past several decades, and one of them is microfluidic technology, with a broad range of applications in cell biology research [81]. A study by De Bem et al. used the endometrium-on-a-chip microfluidics approach, which can mimic the bovine endometrium in vitro, to study the transcriptome and proteomic secretome of endometrial cells (epithelial and stromal cells) under the influence of maternal metabolic factors [82]. It has been observed that metabolic factors such as glucose and insulin alter the transcriptome and protein secretome content of the uterine luminal fluid. In a transcriptomics study, 21 genes in epithelial and 191 genes in stromal cells were altered under high glucose concentrations, while there was limited change in gene expression under the influence of insulin. In a quantitative proteomics study, 1 and 23 proteins were altered in epithelial and stromal cells, respectively, under the presence of glucose, while 196 proteins were found in the secretome under the influence of insulin [82]. The proteins identified in this study were involved in different biological processes; and the pathways which were regulated by different concentrations of insulin were as follows: synthesis of amino acids, carbon metabolism, synthesis of antibodies, metabolic pathways, complement and coagulation cascades, protein processing in endoplasmic reticulum, and amoebiasis, protein digestion and absorption, extracellular matrix (ECM)–receptor interaction, and proteoglycans in cancer (Proteoglycans are present on the cell surface and can interact with both ligands and receptors, thus, execute multiple functions in cancer), while two major pathways like platelet and lysosome pathways were up-regulated by glucose treatment [82]. Altogether, this study demonstrated the use of a novel in vitro model to study uterine functions and fetal-uterine interaction. This study revealed that metabolic factors regulate uterine functions, which might hamper the process of implantation, leading to pregnancy failure.

#### 2.2.3. Proteomics of Exosomal microRNAs in Uterine Lumen Fluid (ULF)

MicroRNAs (miRNAs) are small non-coding RNAs that act as post-transcriptional regulators of gene expression and are present in multiple subcellular compartments, including the rough endoplasmic reticulum, processing (P)-bodies, trans-Golgi network, lysosomes, mitochondria, and nucleus [34]. The miRNAs that are released by the endometrium are known to be involved in the implantation and pregnancy processes, and their abnormal expression is associated with a lack of fetal-maternal cross-talk and pregnancy failure. The miRNAs may serve as a better alternative to invasive biomarkers for analysis of implantation or reproductive health in animals, as invasive methods pose a threat to embryo implantation [83,84,85]. Recently, Kusama et al. used iTRAQ-based analysis to investigate the global protein content and exosomal miRNAs in the UF of cows at Day 7 of pregnancy [85]. A total of 260 proteins were up-regulated among the total identified 336 proteins. These proteins were mainly involved in innate immunity and, more specifically, in neutrophil-mediated immune responses [85]. The isolated exosomes from UF expressed 37 miRNAs, of which 3 miRNAs were low-abundant and six miRNAs were high-abundant in artificially inseminated cows.

Principal component analysis showed that miRNAs and proteins found in UF have a strong relationship and identified a unique protein called suppressor of the G2 allele of the SKP1 homolog (SUGT1), associated with embryonic development [34]. SUGT1 (suppressor of the G2 allele of the SKP1 homolog) is a highly conserved protein involved in multiple biological processes such as innate immune response, centrosome organization, and cytokinesis [86]. Further studies are needed to understand the role of this protein in regulating the uterine environment during pregnancy. In addition to this study, Koh et al. compared the exosomes extracted from the blood plasma of high- and low-fertility heifers using mass spectrometry and identified 4 unique proteins in high-fertility heifers and 31 unique proteins in low-fertility heifers [87]. This study suggested the involvement of these proteins in biological processes specific to fertility. 

The data presented in the proteomics-based studies on endometrial tissues and UF gives an insight into the complex mechanisms of pregnancy, including implantation, embryo development, uterine molecular signatures at the postpartum period, and puberty. However, further investigation is needed to understand the complex process of pregnancy, which can address issues related to reproductive failure in animals.

### 2.3. Proteome Profiling of Oviductal Fluids (OF)

The oviduct is the part of the reproductive tract in mammalian species where several crucial events take place, such as sperm storage, transportation of sperm and oocytes, fertilization, and embryonic development, which are indispensable for a successful pregnancy. Fertilization occurs when oocytes are released by the process of ovulation into the oviduct, which allows the stored sperm to fertilize with the oocytes. The oviduct remains under the influence of different factors and undergoes physiological and morphological changes [88,89]. The oviductal fluid (OF) is secreted by oviductal luminal epithelial cells and is composed of complex mixtures of ions, proteins, metabolites, lipids, and glycans [90,91]. It is essential to understand how the OF affects the mechanisms of fertilization, oocyte-sperm interaction, and early embryonic development. In recent years, several attempts have been made to understand the global contents of OF using “omics”-based studies. 

The proteome analysis of the bovine OF revealed many DEPs according to the site of ovulation, the stage of the oestrous cycle, and progesterone concentration using the label-free method [92]. The OF (oviductal fluid) was analyzed at four stages of the oestrous cycle in cyclic cows: pre-ovulatory, post-ovulatory, mid-, and late-luteal phases. A total of 482 proteins were present on oviductal secretions during the oestrous cycle, and anexin A1 was the most abundant protein at the pre-ovulatory stage, whereas heat shock proteins (HSP) were upregulated at the post-ovulatory bovine ipsilateral OF [92].

Annexins, a family of calcium-dependent phospholipid-binding proteins, are a highly conserved group of proteins present in all eukaryotes, including humans, animals, and plants. There are more than 100 types of annexins discovered so far across all species. The common composition of annexins involves two main domains: the head (NH_2_-terminal) and the core (COOH-terminal). The core region is conserved, while the head is divergent among the members of the annexin family, and the C-terminal interacts with calcium, further mediating canonical membrane binding properties [93,94]. Annexins are involved in different biological processes, including exocytosis, endocytosis, stabilization of the plasma membrane at a specific site, RNA-binding, and nucleotide-binding functions. However, the functions of all annexins are not fully understood [95]. Several annexins are found to be associated with female reproduction and are expressed at different sites of the uterus. Annexins A2, A1, and A7 are expressed by endometrial cells and regulate endometrial receptivity and embryo implantation in humans and mice [96,97,98].

In another study, Pillai et al. used secretions from in vitro models of bovine oviductal epithelial cells (OEC) and ex vivo OF; a total of 2087 proteins were identified, of which 266 were secretory in nature [99]. In terms of functions, proteins were active in immune homeostasis, gamete maturation, fertilization, and early embryonic development. The OF (oviductal fluid) also contains exosomes, which may act as key modulators of embryonic development and survival. Alminana et al. proposed that the exosomes play a vital role in early embryonic development and gamete-oviduct interactions and characterized the molecular signatures of bovine oviductal extracellular vesicles (oEVs) derived from in vivo and in vitro samples at different stages of the bovine estrous cycle using mass-spectrometry-based approaches [100,101]. In the first study, a total of 319 proteins were found in oviductal extracellular vesicles (oEVs) at the post-ovulatory stage. Then, they compared the protein content of in vivo and in vitro oEVs; 186 DEPs were identified in oEVs. This study revealed important proteins that regulate the gamete/embryo-oviduct interactions, which are oviductal glycoprotein 1, HSP90, A8, and 70, gelsolin (GSN), and ezrin (EZR) [100]. In the second study, they analyzed the oEVs at different stages of the bovine estrous cycle. A total of 336 proteins were identified in this study, out of which 170 were differentially expressed across the estrous cycle (*p*-value < 0.05, ratio < 0.5, or >2). GO analysis showed overrepresented terms related to vesicles, focal adhesion, cytoskeleton, cell surface metalloexopeptidase activity, and the innate immune system [101].

In general, OF is obtained through invasive surgery, a labor-intensive and time-consuming process that is stressful for the animals. Papp et al. developed a novel method to retrieve the OF using a less invasive method of transvaginal endoscopy for proteome profiling [102]. In total, 3001 proteins were identified (FDR ≤ 1%) in heifers at two different stages of the estrous cycle (Day 1 and Day 3), and it was observed that common proteins like oviductal glycoprotein 1 and HSP identified in this study have also been reported by previous groups [102]. Comparative analysis of the OF (oviductal fluid) proteome between pregnant and cyclic heifers at different oviductal regions, including the ampulla and isthmus, on Day 3 post-estrus revealed many interesting findings [103]. Some important proteins, such as serum albumin, serotransferrin, stress-induced phosphoprotein 1, phosphoglycerate kinase 1, and serpina1, were in high abundance in the presence of embryos, indicating a potential role in embryonic development [103]. Another study with the bovine OF proteome, taking into account three factors such as the anatomical oviduct region (isthmus vs. ampulla), the peri-ovulatory stage (pre-ovulatory vs. post-ovulatory), and the proximity of the ovulating ovary (ipsi- lateral vs. contralateral side) using the nano-LC-MS/MS approach, identified many DEPs [90]. This study identified a total of 3760 proteins in the bovine OF, which are the most comprehensive proteomes published until now. They performed a quantitative analysis using a label-free proteomic approach and found differentially abundant proteins involved in a broad range of biological functions like protein binding, response to stress, cellular adhesion, calcium homeostasis, and immune system modulation [90]. Many abundant proteins were identified in bovine OF, including several HSPs, myosin, CD109, complement C3, and oviductin, as described in other studies as well. These studies covered various factors that influence the bovine OF, including the stage of the cycle, the presence of gametes, the anatomical region of the oviduct, the sides of ovulation, and the in vitro secretome of the OF. However, there are several other factors, such as fertility index, metabolic stress, and the effects of lactation, that can modulate the proteome of bovine OF, thus affecting embryonic development and early implantation [104]. In an attempt to analyze the effects of fertility index and metabolic stress on dairy cows, Gegenfurtner et al. compared OF from two animal models: a metabolic model (postpartum lactating and non-lactating cows) and a genetic model (low- and high-fertility index) using nano-liquid chromatography tandem mass spectrometry analysis and label-free quantification [104]. A total of 1976 proteins were present in OF, of which, in the metabolic model, a total of 37 significantly abundant proteins were found (*p*-value < 0.05, log2-fold change > 0.6), while in the genetic model, 110 proteins were significantly abundant (*p*-value < 0.05, log2-fold change > 0.6) in pair-wise comparisons. The analysis of GO terms for abundant proteins revealed associations with actin binding, translation, and immune system processes [104].

### 2.4. Proteome Profiling of Cows with Endometritis

Endometritis is a common disease with a major impact on the reproductive health of animals, which burdens the farm industry. The pathogenic bacteria often cause inflammation in the endometrial lining during parturition and coitus [105]. The endometrium plays an important role in reproduction, nourishment of the early embryo, and sperm transit. The inflammation in the endometrium hinders sperm mobility and embryo implantation, leading to pregnancy failure [106]. Postpartum bacterial infections affect 40% of dairy cows worldwide [107]. The endometrium consists of epithelial and stromal cells that express pathogen recognition receptors that show an innate immune response against microbes such as *Escherichia coli*, *Fusobacterium necrophorum*, *Prevotella* species, and *Trueperella pyogenes* (isolated from cows with endometritis) [105,107].

Over the past two decades, extensive research has been performed to identify the biomarkers related to endometritis. Advancements in the field of proteomics have renewed interest in the treatment of reproductive disorders in animals [108]. High-throughput proteomics-based approaches are great tools to explore the plethora of protein signatures associated with endometritis. Various sources, such as endometrial tissues, blood plasma, exosomes, and uterine secretions, have been used to investigate the protein profile associated with endometritis using proteomics approaches like 2-D gel electrophoresis and iTRAQ-based mass spectrometry.

Cairoli et al. investigated the protein expression pattern in cows during pregnancy and the peripartum period, with and without postpartum uterine infection, using a gel-based approach [109]. They identified a high difference in acute-phase proteins, including orosomucoid and haptoglobin, during the last phase of pregnancy and early postpartum. A very high level of orosomucoid was also observed in cows with postpartum endometritis after 2 weeks of calving. Orosomucoid is also known as α-1-acid glycoprotein (AGP), which is an anti-inflammatory protein, and its role is unclear [110]. The acute phase proteins are components of the natural defensive system and play a crucial role in the prevention of infections and the regeneration of tissues. In bovines, haptoglobin and serum amyloid A are found to be associated with reproduction physiology [111]. The acute phase proteins are sensitive and non-specific indicators of inflammation or infection, and recently they have been used as therapeutic targets in veterinary medicine in large animals for restoration of homeostasis [112].

Based on 2D-gel electrophoresis followed by protein identification by mass spectrometry, Choe et al. and Legard et al. compared cows with a normal uterus to cows with an endometritic uterus [28,106]. The former group identified 12 DEPs in endometrium with endometritis, and they suggested that desmin, α-actin, HSP27, HSP70, and MHC-I may play an important role in endometrium for successful implantation [106]. The latter group identified 12 proteins with high abundance in cows infected with *Trueperella pyogenes* in comparison to normal cows [28]. They also compared protein expression in relation to the percentage of polymorphonuclear neutrophils (PMNs) (0–35% PMNs) in lactating cows at 15 and 42 days postpartum [28]. The PMNs are the first line of defense and are released in the uterus after a pathogen attack. The detection of PMNs in uterine samples is frequently used as a diagnostic tool for the identification of uterine health. Few indicator proteins were found in the study that had a positive correlation with the percentage of PMNs (>10% PMN), namely cathelicidin, peptidoglycan recognition protein 1, serine protease inhibitor B1, and calprotectin at 15 days postpartum. The expression of these proteins was high in cows with an increased presence of *T. pyogenes* and an elevated PMN percentage [28].

A study with the in vitro model of bovine endometrial epithelial cells (bEECs) to study the effect of *E. coli* lipopolysaccharide (LPS) by 2D-gel electrophoresis coupled with MALDI-TOF/TOF mass spectrometry revealed 38 DEPs (*p* < 0.05 to *p* < 0.001), of which 28 were up-regulated and 10 were down-regulated under the exposure of LPS [113]. These DEPs were involved in processes like apoptosis, cell structure organization, regulation of histones, and various other metabolic pathways. Studies have identified different sets of proteins related to endometritis [114,115]. Using more advanced proteomics techniques like iTRAQ proteomic analysis, Zhang et al. identified 159 and 137 DEPs in the endometrium and blood plasma, respectively, in cows with endometritis in comparison to normal cows [114]. The functional analysis of DEPs indicated that extracellular hydrolase activity was higher in tissues, causing inflammatory damage, which may account for the provocation of endometritis. Using a similar technique, Ren et al. analyzed the effects of prenatal supplementation with selenium and yeast in postpartum dairy cows [115]. A total of 542 proteins were identified in plasma extracted from calving cows, out of which 48 were DEPs (with a fold change ≥ 1.2 or ≤0.83; *p* < 0.05). The bioinformatic analysis of DEPs indicated the involvement of proteins in acute phase reactions, positive regulation of lipid metabolic processes, and defense responses to bacteria or fungi [115]. It is suggested that the low abundance of proteins involved in acute-phase reactions indicates that selenium reduces systemic inflammation after calving.

Miller et al. performed a comparative quantitative label-free proteomic analysis on the blood plasma of dairy cattle with cytological endometritis and without endometritis [116]. A total of 25 proteins were differentially expressed out of 181 total proteins identified in these samples. This study provided a potential protein marker, “uncharacterized protein G5E513”, in the plasma of cows affected by endometritis [116]. Until now, endometrial tissues and blood plasma were used to explore the proteome related to endometritis. Uterine secretions are rich in different factors that affect reproductive health and have been extensively used for proteomic analysis. Helfrich et al. used uterine secretions for the analysis of subclinical endometritis in cattle, and the proteome profiling of uterine secretion revealed a plethora of proteins, with some of them involved in inflammation and immune defense mechanisms [117]. In addition to these sources, exosomes have also been utilized to understand endometritis in dairy cows.

Over the past decades, exosomes have gained immense attention from researchers worldwide for their potential to understand the cellular and molecular mechanisms of diseases, cell-cell communication, and homeostasis. In addition to that, exosomes are also potential therapeutic tools for the treatment of various diseases. Exosomes are small endosomal-derived membrane microvesicles measuring 40–100 nm in diameter. Based on their origin in cells, exosomes are composed of proteins, nucleic acids, lipids, amino acids, and metabolites. Exosomes also play a role in the immune response during infections [118,119,120]. However, the role of exosomes in relation to endometritis is not fully understood. Almughlliq et al. compared the exosomes isolated from infected and non-infected cows using high-performance liquid chromatography tandem mass spectrometry [121]. In the exosomes of infected cows, 103 bovine and 9 *Trueperella pyogenes* proteins were present with 71 unique bovine proteins, whereas in normal exosomes, 90 bovine and 5 *T. pyogenes* proteins were found with 4 unique bovine proteins [121]. The protein cargo of the infected exosomes was involved in immune system processes and unique pathways, including angiogenesis and the integrin signaling pathway. This study revealed that pentraxin, a unique protein in the infected exosomes, might be a potential candidate as a biomarker for uterine infection diagnosis [121]. Pentraxins are a superfamily of multi-functional conserved molecules consisting of a pentraxin domain at the C-terminal and act as mediators of immune system regulation. This family has been broadly categorized into two groups: short pentraxins, which compromise mainly C-reactive protein and the serum amyloid P component, and long pentraxins (neuronal pentraxin 1 and 2, neuronal pentraxin receptor, pentraxin 3 and 4) [122,123].

## 3. Proteomics of the Sheep Uterine Milieu to Unfold the Molecular Landscape of Implantation

Sheep is a compelling model to investigate the physiological, molecular, and biochemical events during embryo-endometrium interaction at the time of implantation and has been proposed as an alternative model to explore cow placental biology due to similarities in the initial formation of the syncytial trophoblasts of cow placentae. Sheep are easy to manipulate, are less expensive, and also present unique characteristics of pregnancy in comparison to rodent and primate models [124]. Similar to bovine, sheep endometrium contains luminal epithelium, glandular epithelium, and stroma, which together regulate the process of pregnancy. The endometrium of sheep has two parts that are essential for the establishment of pregnancy: the aglandular caruncular (C) part, which provides a site for superficial implantation, and the glandular intercaruncular (IC) part, which plays a role in the synthesis and secretion of histotroph [37]. However, timing and length of implantation differ among species due to the difference in implantation phases (hours in rodents to days in humans and domestic animals), pattern of endometrial invasion by the trophoblast, and cell–cell contact [3]. The research on animal models provides essential information related to molecular and physiological aspects of pregnancy, which will help in designing therapeutic strategies for the treatment of infertility and pregnancy failure [38]. Similar to bovine endometrial tissues, UF and exosomes are investigated using proteomic techniques (Table 1) to unfold the molecular landscape oriented to embryo implantation in sheep.

### 3.1. Proteome Profiling of Uterine Fluids (Pregnant vs. Non-Pregnant)

The proteomic analysis was performed by Koch et al. to identify the protein signatures in sheep uterine lumen by using LC-MS/MS analysis on uterine fluid from nonpregnant vs. early-pregnant animals [41]. In early pregnant sheep, 15 proteins related to embryonic development were found, of which specific uterine remodeling proteins (transgelin) and placental proteins (PP9) were highly abundant in comparison to nonpregnant sheep [41].

### 3.2. Proteome Profiling of Endometrial Tissues (Intercaruncular vs. Caruncular Areas)

Adding more information to sheep proteome datasets in relation to early pregnancy, Wang et al. compared sheep endometrial C (caruncular) area vs. IC (intercaruncular) area on Day 17 of pregnancy and observed 170 DEPs related to growth and remodeling of endometrial tissue and cell adhesion [37]. Like in the previous study, transgelin was up-regulated in the C area, supporting its role in uterine remodeling. Furthermore, the proteome profiles of C and IC on Days 12 and 16 of the oestrous cycle and at three stages of pregnancy (conceptus pre-attachment, implantation, and post-implantation) revealed the proteins associated with protein synthesis and degradation, antioxidant activity, cell structural integrity, cellular adhesion, and signal transduction [37]. The proteins including tryptophanyl tRNA synthetase (WARS, member of the aminoacyl-tRNA synthetase family), endoplasmic reticulum resident protein 57 (ERP57, member of the protein disulphide isomerase family), mitochondrial manganese superoxide dismutase 2 (SOD2, antioxidant scavenging enzymes), and vimentin (VIM, member of the intermediate filament family) were upregulated in caruncular tissue at the time of implantation and during early pregnancy [38]. The presence of an embryo also alters the proteome of the intercaruncular endometrium during early pregnancy. A study by Arianmanesh et al. showed DEPs in caruncular endometrium from the gravid and the nongravid uterine horns at Day 16 and Day 20 of pregnancy [40]. Fifty-seven and twenty-seven proteins were up-regulated on Day 16 and Day 20 of pregnancy, respectively. This study suggested the role of adenosyl homocysteinase (AHCY), carbonic anhydrase (CAII), HSP60, and apolipoprotein A-1 (APOA1) during conceptus implantation and the early post-implantation period [40].

In another study with the caruncular and intercaruncular areas of pregnant and non-pregnant sheep using LC-ESI-MS/MS with LTQ-orbitrap collision-induced dissociation (CID), 94 and 257 DEPs were revealed in the endometrial caruncular and intercaruncular areas, respectively [39]. The DEPs were generally associated with proteasome-mediated proteolysis, immune response, and nutrient transport and utilization. The variation in expression patterns of extracellular matrix (ECM) proteins was observed between the IC and C areas. The high abundance of ECM proteins and focal adhesion proteins indicated their essential role in maternal–fetal interaction [39].

Fecundity critically affects the profitability of sheep production, but the molecular mechanism of high prolificacy is still unclear [11,125]. Previous studies have utilized a proteomics-based approach to reveal the factors that contribute to high prolificacy in sheep in ovary tissue and uterine tissue. Some important pathways, such as ribosome assembly, the mTOR pathway, energy metabolism, hormone synthesis, ovarian function, sphingolipid metabolism, and amino acid metabolism, were prominent in sheep in relation to high prolificacy [9,125,126].

### 3.3. Proteome Profiling of the Genital Tract (Day 0 vs. Day 10 of the Oestrous Cycle)

Soleilhavoup et al. described the integrated analysis of the luminal proteomes of the sheep genital tract, i.e., the inner cervical mucus, uterine fluid, and oviductal fluid, and quantified the abundant luminal proteins at two points of the oestrous cycle (Day 0 versus Day 10) [127]. A total of 749, 827, and 624 proteins were found in the cervix, uterus, and oviductal fluids, respectively, and 291 proteins were differentially expressed throughout the cycle in fluids from different regions [127]. At Day 0, HSP, mucins, complement cascade proteins, redox enzymes, and gamete-interacting proteins such as oviductin, osteopontin, HSPA8, and spermadhesin were overexpressed, whereas, at Day 10, proteins of the immune system (ceruloplasmin, lactoferrin, Deleted in Malignant Brain Tumors 1) and tissue remodeling proteins (galectin 3 binding protein, alkaline phosphatase, CD9, or fibulin) were overexpressed [127].

### 3.4. Proteome Profiling of Uterine Luminal Fluid (Days 12 to 16 of Pregnancy)

Brooks et al. provided more insight into the proteome of uterine luminal fluid as pregnancy progressed from Day 12 to 16, along with the transcriptome analysis of the uterine luminal epithelium (LE), glandular epithelium (GE), and conceptus [41]. More than 400 proteins were found in the ULF, and the number of proteins increased from Day 10 to Day 16 (79 proteins on Day 10, 102 proteins on Day 12, 167 proteins on Day 14, and 201 proteins on Day 16) involved in cellular reorganization, protein folding, and many other cellular processes [42]. A study by Romero et al. gave a comprehensive picture of the uterine environment in the establishment of pregnancy between Days 12–16 by analyzing UF from Day 12 of the estrous cycle (non-pregnant) and from Days 12, 14, and 16 of pregnant sheep and unraveling the molecules necessary for the establishment of pregnancy [43]. A total of 783 proteins were present from Days 14–16 of pregnancy in UF, of which 7 proteins, namely annexin A1, A2, and A5, calcium-binding protein (S100A11); profilin 1; trophoblast kunitz domain protein 1 (TKDP); and interferon tau (IFNT), were involved in inhibition of prostaglandins (annexins and S100A11); protecting against maternal proteases (TKDP); cytoskeleton remodeling (profilin 1); and monitoring the release of prostaglandin F2α (IFNT). The expression of IFNT increased from Day 14 to Day 16 in the UF of pregnant ewes, confirming its essential role in maternal recognition of the pregnancy signal [43]. Unique proteins, COL1A1 and COL1A2, from monomeric type 1 collagen, were detected in UF during the estrous cycle as compared to the UF of pregnancy on Day 12 [43].

### 3.5. Proteome Profiling of Conceptuses and Endometrium

On a quantitative comparison by Yang et al. between in vivo and in vitro conceptuses and their corresponding endometrial caruncular (C) and intercaruncular (IC) areas on Day 17 of pregnancy during the peri-implantation period, it was found to have impaired metabolic pathways and redox homeostasis in in vitro conceptuses, which might hinder the endometrial receptivity due to inappropriate reciprocal communication [128]. The corresponding area of caruncular and intercaruncular tissue in the in vitro conceptuses showed an aberrant abundance of proteins such as ECM-interacting proteins, endometrial remodeling proteins, and redox homeostasis-related proteins [128]. The researchers observed a new finding in their study: impaired methyl metabolism in in vitro conceptuses, which provides methyl groups for methyl transfer reactions in the DNA methylation process [128]. DNA methylation is an epigenetic mechanism that controls gene expression by regulating the transcription factors binding to DNA. Through de novo DNA methylation and demethylation, the pattern of DNA methylation in the genome changes during development in a tissue-specific manner, which regulates gene transcription during development. DNA methyl transferases (DNMTs) maintain the methylation profile of genes, and the aberrant expression of DNMTs in IVF embryos has shown growth and developmental arrest in IVF embryos [129,130].

In a very recent study, Yang and co-workers demonstrated a comprehensive proteomic map of ligand–receptor pathway cascades essential for maternal–fetal interaction at the protein level [131]. The differential proteomic analysis revealed several potential ligand–receptor pathway cascades at the maternal–fetal interface. This research group screened the differentially abundant proteins across the conceptus and endometium and constructed a repository of potential ligand–receptor pathway cascades [131]. They found overexpression of the membrane protein calpain 2 (CAPN2) in endometrial tissues and its interacting secreted protein cathepsin B (CTSB) in the conceptus. The membrane proteins fibronectin 1 (FN1) and nucleoside diphosphate kinase 1 (NME1) were enriched in the conceptus, while their interacting secreted partners, integrin subunit beta 1 (ITGB1) and FK506 binding protein 1A (FKBP1A), respectively, were enriched in the endometrial tissues. The researchers also identified some putative interactions that might contribute at the maternal–fetal interface, including conceptus-secreted and endometrial membrane partners (CD109-ALPL, PRXL2A-HSP90AB1, and fibronectin 1-protein tyrosine kinase 7), as well as endometrium-secreted and conceptus-enriched partners (ALB-CLDN4, DCN-AGRN, and GC-LRP2) [131]. These cascades were associated with cell adhesion and invasion, redox homeostasis, and the immune response. The researchers suggested a novel function of enhanced glycolysis in endometrial remodeling and the production of lactate during glycolysis, inducing lactylation in histone protein H3K18 [131].

### 3.6. Proteome Profiling of Exosomes 

The exosomes from endometrial cells play an important role during implantation [132]. The exosomes contain molecules like miRNAs, mRNAs, and proteins [133]. The miRNAs regulate gene expression, and thus exosomes can elicit biological effects during fetal development [134]. A proteomic study by Burns et al. showed that endogenous beta retroviruses (enJSRVs), which are expressed in sheep endometrial tissues, are transferred to the conceptus via exosomes. The enJSRVs Env is an essential component for conceptus elongation in sheep [134]. The extracellular vesicles are also released by the conceptus, which might also be involved in maternal-fetal communication. In proteomic analysis of extracellular vesicles released from the conceptus, 231 proteins were enriched for extracellular space, and several proteins belonged to proteases, protease inhibitors, chaperones, and chaperonins [134]. Comparative proteome analysis of extracellular vesicle proteins was conducted by O’Neil et al. using extracellular vesicles from Day 14 cyclic and pregnant sheep [132]. Key exosomal protein markers (Alix, TSG101, CD63, HSP70, CD9, and CD81) were identified in exosomes [104]. Proteins like alkaline phosphatase (ALPL), gag (Gag), an uncharacterized protein (LOC105602254), mucin 16 (MUC16), and milk fat globule-EGF factor 8 (MFGE8) were overexpressed in cyclic and pregnant sheep. Further in vitro analyses showed decreased ovine trophectoderm cell proliferation and increased IFNT production.

## 4. Proteomics of Pigs Uterine Milieu to Understand the Mechanism of Implantation for Successful Pregnancy 

Pigs are economically important animals in terms of pork production, and thus the efficiency of production depends on a successful pregnancy. The embryonic mortality rate is around 20–30% in pigs in the first 30 days (peri-implantation) of pregnancy, and additionally, 10–20% of embryos are lost in the 85 days (mid-to-late gestation in pigs) of pregnancy. However, the cause of the embryonic mortality is not clear [135,136]. The mechanism of implantation and placentation is different in pigs as compared to rodents and sheep because pigs have a unique, non-invasive epitheliochorial placenta in which the uterine luminal epithelium (LE) is intact throughout pregnancy. In pigs, the regulation of uterine functions during the early phase of pregnancy is unique, where estrogen is the key factor of pregnancy recognition, while in bovines and sheep, interferon tau is a major factor in maternal recognition of pregnancy [44,137]. On Days 11 and 12, the trophectoderm of the pig conceptus differentiates and expands, and this period is called the maternal recognition of pregnancy. During this period, an enhanced level of estrogen induces the secretion of many factors from the uterus that are critical for implantation [45,138]. The pigs also serve as a valuable model to study pregnancy-related protein expression using proteomic approaches and have gained attention in the fields of biomedical and agricultural sciences [139]. Thus, proteomic approaches provide a better understanding and characterization of proteins involved in molecular and biological processes in pigs [47]. There are very few studies that describe the proteome profile of the early phase of pregnancy in pigs (Table 1).

### 4.1. Proteome Profiling of Oviductal Fluid and Conceptus 

For the first time, Georgiou et al. applied high-resolution 2D gel electrophoresis together with mass spectrometry to analyze the changes taking place in the protein contents of oviductal fluids in the presence of oocytes and sperm. It was observed that 19 and 4 proteins were affected by the presence of sperm and oocytes, respectively, and these proteins were involved in protein synthesis, maintenance, and repair (41%); antioxidant and free radical scavenger metabolism (18%); cellular metabolism (15%); and miscellaneous processes (25%) [140]. For adequate cross-talk between the conceptus and endometrium, the elongation of the conceptus, i.e., the increased surface area of the trophectoderm for attachment to the uterine surface epithelium, is essential, and during this phase, the chances of embryo loss are higher [138]. Degrelle et al. identified 174 abundant proteins in the conceptus in a stage-specific manner during elongation at the time prior to implantation. Proteins such as ezrin (EZR), tropomyosin 3 (TPM3), annexins (ANXA5, and A8), and keratins (KRT8, 18, and 7) were involved in cell proliferation/differentiation, cytoskeletal organization, cellular metabolism, and stress response [138].

### 4.2. Proteome Profiling of Endometrial Tissues (Pregnant vs. Non-Pregnant) 

A comparison between pregnant and non-pregnant pigs at days 40, 70, and 93 of pregnancy revealed 98 differentially abundant proteins in endometrial tissues [44]. Some of the important protein categories detected were cytoskeletal proteins (septin 2, stathmin 1, cofilin 1, and fascin1), development- and cell death-associated proteins (transferrin, protein DJ-1, transgelin, and galectin 1), and chaperones and anti-apoptotic proteins (HSP 90a, HSP 90b, HSP 70, HSP 60, and HSP 27) [44]. The early phase of pregnancy, specifically the second and third weeks of gestation in pigs, is critical for a successful pregnancy and has a direct influence on litter size. The study by Jalali et al. identified several proteins in pregnant pigs in comparison to cyclic pigs on Days 9 and 12 [45]. The proteins essential for the establishment of pregnancy, including annexin A4, beta-actin apolipoprotein, ceruloplasmin, and afamin, were abundant on Days 9 to 12 of the estrous cycle, which help in preparing the endometrium to receive the embryo, while conceptus-released factors such as haptoglobin, prolyl-4-hydroxylase, aldose-reductase, and transthyretin (TTR) were also over-expressed in pregnant pigs on Day 12 [45]. Some pregnancy-related pathways were also identified, such as acute phase response, LXR/RXR activation, regulation of actin-based motility, and IL-12 signaling. Transthyretin (TTR), haptoglobin, and cyclophilin A have been suggested as potential biomarkers for human embryo receptivity [45]. However, none of these studies give a picture of the cellular and molecular changes occurring in the endometrium in the presence of the conceptus. Jalali and co-workers in another study showed the essential protein cargo present in the endometrium in the presence of conceptus on Day 12 of pregnancy, which includes serpins, cofilin, annexin A2, aldose reductase, cyclophilin, protein disulfide isomerase A3, and peroxiredoxin 1 [46]. The presence of embryos activates several pathways, such as calcium signaling, tissue remodeling, immune modulation, and antioxidant defense systems, which are essential for endometrium receptivity.

### 4.3. Proteome Profiling of Endometrial Tissue (Preimplantation and Peri-Implantation Period)

Adding more information about molecular mechanisms related to uterine receptivity in pigs, Pierzchała et al. employed a gel-based proteomics approach and MALDI-TOF/TOF mass spectrometry to determine the proteome of the uterine endometrial tissue during the preimplantation and peri-implantation periods of pregnant pigs between Days 9, 12, and 16 [47]. A total of sixteen DEPs were identified, mostly involved in the ECM organization (actin; ACTR3), cytoskeletal organization (ACTB, CAPZB), cellular lipid metabolism (APOA1, TTR), early phase of apoptosis (ANXA4), maternal immunomodulation (HP), angiogenesis (LDHB), and thyroid hormone secretion (CRYM; TTR), indicating their role in modulation of endometrial receptivity and embryonic development [47].

### 4.4. Proteome Profiling of Luminal Fluid

Furthermore, He et al. used iTRAQ-based proteomics technology to identify and analyze pig uterine luminal fluid proteins on Day 9 of the oestrous cycle and Days 9, 12, and 15 of pregnancy and detected a total of 964 proteins [141]. Out of the total identified proteins, 279 were differentially abundant; 5 clusters of proteins were generated using self-organizing tree algorithm (SOTA) clustering that were involved in some important processes such as regulation of low-density lipoproteins and TGF-β secretion [141]. It is suggested that the regulation of TGF-β is important for pig embryonic morphological transformation. The researchers also identified proteins that play a role in implantation, such as cathepsin C and B (CTSC and CTSB) and acid phosphatase 5 (ACP5) [142]. ACP5 is also known as tartrate-resistant acid phosphatase or uteroferrin, which is a metalloprotein expressed by the endometrial glandular epithelium of mammals, including cows, sheep, goats, and pigs [142]. Uteroferrin plays a crucial role in iron transport from the maternal-to-fetal compartment and also promotes the uptake of macromolecules [143].

### 4.5. Proteome Profiling of Serum 

The analysis of the serum proteome in the pig during early pregnancy on Days 0, 5, 12, 16, and 19 of gestation using data-independent acquisition (DIA) mass spectrometry with verification by parallel reaction monitoring (PRM) found 113 DEPs that were implicated in catalytic activity, metabolic processes, and proteasome function. Four DEPs, such as talin 1, profilin, carbonic anhydrase, and hepatocyte growth factor (HGF) activator, were detected by both DIA and PRM [144].

### 4.6. Proteome Profiling of Endometrial Tissue (High Prolific Breed vs. Low Prolific Breed)

The litter size is important in pigs in terms of the economy of the farm industry, which is affected by factors like uterine capacity, ovulation rate, and survival of embryos [48,145]. However, there are few studies that have made efforts to describe the molecular basis for embryo loss during the mid-gestation period in pigs. Wang et al. have analyzed the protein expression in pigs with regard to fetal loss in mid-gestation using the iTRAQ technique [48]. The quantitative protein profiling between the high prolific breed (Meishan pig) and low prolific breed (Duroc pig) during mid-gestation (Days 49 and 72) revealed several DEPs in Duroc pig, which were associated with many metabolic processes, including arginine metabolism. Arginine is essential for embryonic survival and fetal development, and it also participates in muscle tissue development [48,146]. Some cytoskeleton-related proteins, such as keratin 18 (KRT18), cardiac muscle alpha actin (ACTC1), desmin (DES), synemin (SYNM), crystallin α B (CRYAB), and peripherin (PRPH), were also over-expressed in Meishan pigs on Day 72 in comparison to Day 49. These intermediate filament proteins are involved in the elongation of the conceptus and the adaptive response of the uterine wall to the increasing mechanical forces due to fetal growth [48]. In relation to endometrial receptivity, three clusters of ECM-remodeling proteins, i.e., extracellular matrix components (COL4A2, COL4A1, LAMB1, LAMB2, TGFBI, HAPLN1, COL3A1, CLU, and FBN1), proteases (CTSB, CTSC, and CTSZ), and protease inhibitors (SLPI, ITIH1, ITIH2, UFAP, UFBP, and SERPING1), were observed in this study. These findings demonstrated the functions of clusters of proteins in various activities of pregnancy, and these proteins might be new targets for improvement of the implantation process and embryonic development.

## 5. Key Signaling Pathways Associated with Early Pregnancy in Farm Animals

In a biological process, proteins do not act as a single entity; rather, they make connections with other proteins to generate a network. The intracellular pathways are inter-connected through complex molecular interactions and are regulated by endocrine, paracrine, or autocrine signals through their receptors. Numerous such signaling pathways are activated during different phases of pregnancy. The dysregulation of these pathways can lead to pregnancy failure [147]. Several important pathways have been identified in studies taken in this review related to early pregnancy, which include TGF-beta signaling pathways [35,91], platelet activation and lysosomal pathways [82], focal adhesion [38,101], Ras signaling pathway and Wnt signaling pathway [101], EGFR, ERBB2, and PI3-kinase (PI3K) pathways [91], antigen processing and presentation and leukocyte transendothelial migration [32], ribosome and cell adhesion molecules (CAMs) [68], IFN signaling, ECM-receptor interaction [38,125], cytoskeletal organization [32,139], and glycolysis/gluconeogenesis pathways [32,140].

Focal adhesions (FAs) are key structural elements that interact with the ECM and include integrins, vinculin, paxillin, talin, and actopaxin. FAs are involved in many cellular processes, such as proliferation, cell polarity, differentiation, anchorage-dependent survival, invasion, migration, and spreading [148]. FAs regulate cellular processes via focal adhesion kinase (FAK), a nonreceptor kinase that is recruited by integrin and subsequently activates other proteins of focal adhesions [149]. Cytoskeleton organization is a crucial process during implantation, as it facilitates differentiation of the uterine epithelial cells [32,139]. In addition, actin and actin-binding proteins are cytoskeletal proteins that transform the cell’s morphology and are crucial for trophoblast adhesion and invasion [139]. The TGF-beta signaling pathway is also activated during the trophoblast invasion and helps in maintaining immune tolerance during embryo-uterine interaction [35,101]. These published data show that these pathways are activated during embryo-endometrial cell interaction and play an important role in the regulation of implantation; however, there is a need for further research on how these pathways are interconnected and regulate the complex process of implantation.

In this review, detailed analysis of these proteomic data taken from different species (bovine, sheep, and pig) showed that the set of identified proteins are different with little overlapping among species. We assume that these variations arise due to various factors: (1) source of samples that is endometrial tissue, uterine luminal fluid, or oviductal fluid; (2) proteomics approaches (gel-based and MS-based; label- or label-free); and (3) conditions and timing for sample collection (different days of early pregnancy and pregnant vs. non-pregnant). The differentially expressed proteins found in tissue and luminal fluids from these studies are summarized in Table 2 for these species. This table shows the differences in the expression patterns of different sets of proteins among species, and common proteins are highlighted in bold. Proteins such as actin and annexins are present in all species, are part of cytoskeletal proteins, and regulate endometrial receptivity during implantation. Studies in humans have shown the association of annexin 5 with spontaneous miscarriage [55]. However, the list of non-overlapping proteins is vast in comparison to common proteins. The studies on these farm animals are not only relevant to animal sciences but also add valuable information for complex biological mechanisms of implantation in humans.

**Figure 3:**
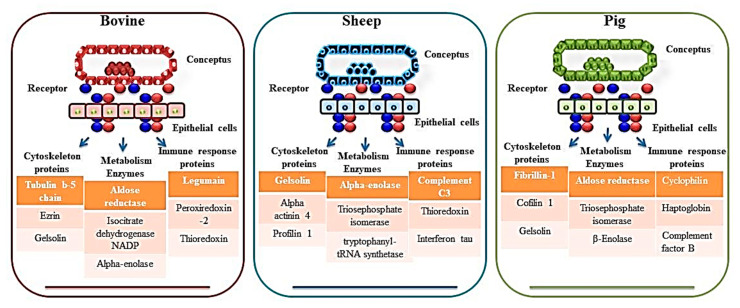
Differentially expressed proteins have been identified by proteomic studies. This illustration represents the differentially expressed proteins identified by different studies and that were present in abundance in endometrial tissue and/or luminal fluids during the early pregnancy of ruminants. All the DEPs presented in this illustration are present in abundance among the different studies. Some of the proteins are common among the species, including ezrin, gelsolin aldose reductase, and enolase, which play an essential role in implantation.

The proteomic studies in farm animals reviewed here provide very useful information about major proteomic changes occurring during the transformation of non-receptive endometrium to receptive endometrium, uterus health, and early embryonic development (Figure 3).

These data give an insight into the expression patterns of these proteins and demonstrate that an aberrant expression of these proteins may impair the uterine environment, further causing infertility in farm animals. Some proteins investigated in endometrial tissue and UF by these proteomic studies have revealed their critical role in early pregnancy (Table 3).

These studies from different farm animals demonstrated that several factors contribute to embryo implantation during early pregnancy. Researchers from different laboratories used various proteomics techniques to decipher the molecular landscape of early pregnancy at different time points. Thus, a large amount of proteomic data are now available in the public domain. Despite the availability of large amounts of data and extensive research, they do not fully reveal the molecular complexity of the embryo-uterine interaction. Different biological sources were used in these studies, and different sets of proteins/proteoforms were identified. Therefore, there is a need for the identification and functional analysis of the most relevant proteins with a major impact on the implantation process. Implantation is a very complex mechanism that is influenced by various factors, including hormones, growth factors, and regulatory proteins. Further studies are required to identify the most relevant proteins/proteoforms associated with the early pregnancy/implantation process and establish the markers of implantation to adopt therapeutic strategies against implantation failure.

## 6. Future Perspectives 

Livestock animals like cows, buffalo, sheep, and pigs contribute substantially to the economy of the nation in terms of milk and meat production. Reproductive health and welfare of the animals should be the priority of livestock owners for optimum productivity. Failure of embryo implantation is a major cause of unsuccessful pregnancy in farm animals, and it causes huge economic loss to the livestock owners. Understanding the in-depth physiological processes governed by signals from the uterine endometrium and the embryo to be implanted will pave the way for future research on designing therapeutics for successful embryo implantation and pregnancy. Proteins are the major molecules in the form of enzymes, regulatory molecules, transcription factors, growth factors, cytokines, and hormones that guide the peri-implantation phenomenon. Thorough knowledge of the proteome signatures during the peri-implantation period will substantially aid in developing strategies to overcome the implantation failure. Moreover, the immunological rejection of the semi-allogenic embryo by the mother should also be studied, which accounts for implantation failure and other pregnancy-related animal and human diseases. The prevalence of immune cells, like natural killer cells and macrophages, in the early pregnancy period and their relevance to successful implantation and pregnancy should also be studied in detail with clues from proteome information. The present review article, with substantial proteome information in this regard, lays the foundations for future studies in veterinary medicine and livestock science, with a focus on the discovery of biomarkers to improve fertility rates in livestock animals. 

## 7. Conclusions

**Figure 4:**
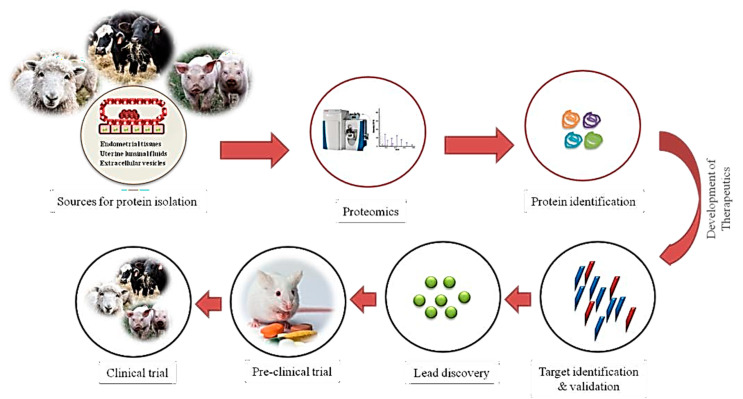
A graphic representation shows the importance of the proteomics approach for novel therapeutic strategies against implantation failure and infertility in livestock animals.

In this review, we summarized the current knowledge of proteomes from different biological sources, such as endometrial tissues, luminal fluids, plasma, exosomes, and oviductal fluids, to understand the involvement of crucial proteins in the embryo implantation of farm animals. These data provide valuable information for the understanding of hierarchy, the sequence of molecular events, and the discovery of novel signature pattern(s) for the early pregnancy uterine environment. Some important pathways and processes were identified from these sources, including cytoskeleton-related proteins, immune system responses, metabolic enzymes, cytokines, and so on (Figure 3). Although a number of significant abundant proteins/proteoforms were found in these studies, there is still a need for further studies to address several issues related to implantation and develop therapeutic strategies to treat implantation failure in farm animals (Figure 4).

## Figures and Tables

**Table 1:** Summary of proteomic reports on early pregnancy in ruminants.

Animal Species	Biological Sources	Gestation Days	Proteomic Approach	Key Findings	References
Bovine	Uterine fluids	Days 16 and 18	2-D gel	9 protein spots were identified in pregnant	[27]
			electrophoresis	compared to non-pregnant	
		Days 5 and 9	2-D gel	10 proteins were differently abundant	[28]
			electrophoresis	between Days 5 and 9	
		Days 7 and 13	Label-free	5 proteins were more abundant on Day 7	[29]
			LC−MS/MS	compared with Day 13, and 29 proteins were	
				more abundant on Day 13 compared with	
				Day 7	
		Day 7 of the	iTRAQ proteomics	35 proteins were up-regulated and 18 were	[30]
		oestrous cycle		down-regulated	
		Days 7 and 15	iTRAQ proteomics	20 proteins were up-regulated and 20 were	[31]
				down-regulated	
		Days 10, 13, 16	iTRAQ proteomics	1652 peptides were identified on day 16	[32]
		and 19			
		Day 7	LC–MS/MS	1563 proteins were detected, of which 472 had not	[33]
				been previously reported	
		Day 7	iTRAQ proteomics	336 proteins were identified, of which 260	[34]
				were more than 2-fold higher in AI	
				cows than Ctrl cows	
	Endometrial	Day 27 after AI	2D-gel	93 protein spots were differentially expressed	[35]
	tissue		electrophoresis	between normal caruncles (NC) and retarded	
			and MALDI-	caruncles	
			ToF/ToF		
		Day 18	2-D DIGE	4 proteins with significantly higher	[36]
				abundances in each sample derived from the	
				pregnant animals	
Sheep	Endometrial	Day 17	LC-MS/MS	170 differentially expressed proteins (DEPs)	[37]
	tissue			were identified. 60 proteins were up-	
				regulated in caruncular areas, and 110 proteins were up-regulated in intercaruncular areas.	
		Days 12, 16, and 20	2DE gel electrophoresis	11 proteins in the caruncular endometrium and six proteins in the intercaruncular endometrium were identified	[38]
		Day 17	LC-MS/MS	94 and 257 differentially expressed proteins (DEPs) were identified in the endometrial caruncular and intercaruncular areas, respectively	[39]
		Days 16 and 20	Two-dimensional gel electrophoresis and mass spectrometry	57 protein spots were up-regulated in the gravid horn at day 16, and 27 protein spots were up-regulated in the gravid horn at day 20	[40]
	Uterine fluids	Day 16	LC-MS/MS	100 of the most abundant uterine luminal proteins were identified, of which 15 were significantly altered in early pregnancy	[41]
		Day 10, 12, 14, 16, and 20	LC-MS/MS	more than 1400 proteins were detected	[42]
		Day 14–16	LC-MS/MS	783 proteins were present by Days 14–16	[43]
Pig	Endometrial tissue	Days 40, 70, and 93	2-DE gel electrophoresis	63 of the 98 proteins are regulated differentially among non-pregnant and pregnant tissues	[44]
		Day 9 to Day 12	2D-DIGE	From a total of 1280 matched spots, 85 spots significantly changed in abundance with the progression of pregnancy from Days 9 to 12	[45]
		Days 11 to 12	2-DE gel electrophoresis	Forty-four differentially abundant proteins in the pregnant endometrium were identified by mass spectrometry	[46]
		9 days (9D), 12 days (12D), and 16 days	2DE-MALDI-TOF/TOF	A total of sixteen differentially expressed proteins (DEPs) were identified	[47]
		Mid-gestation day 49, day 72	iTRAQ	A total of 2170 proteins were identified, and 114 differentially expressed proteins (DEPs) were identified in Meishan and Duroc sows, respectively	[48]

**Table 2:** Differentially expressed proteins were identified among different species using proteomics tools at different days of early pregnancy, i.e., pregnant vs. non-Pregnant animals and different days of pregnancy.

**Pregnant vs. Non Pregnant; Days of Pregnancy (Days 12, 16, 18)**			**References**
Bovine	Endometrial Tissue	Luminal Fluid	
	Rho GDP dissociation inhibitor beta, 20 alpha-hydroxysteroid dehydrogenase (20 alpha-HSD), NADP1-dependent isocitrate dehydrogenase 1, acyl-CoA-binding protein, HSP 90-alpha, calreticulin, annexin A1, annexin A2, fibrinogen alphachain, alpha-1-antiproteinase, serpin H1, serpin A3-8), nuclear ribonucleoproteins A2/B1 and K, serine/threonineprotein phosphatase 2A, ezrin, amine oxidase-A, haptoglobin, albumin, serotransferrin precursor (TF), ovalbumin, isocitrate dehydrogenase, cytoplasmic (IDHA), purine nucleoside phosphorylase (PNP), cystatin-M precursor (CST6), retinol-binding protein 4, aldose reductase (ALDR), cathepsin D (CATD), heat-shock cognate 71 kDa protein (HSP7C), actin, cytoplasmic 1 (ACTB), prepro complement component C3 (CO3), cathepsin B precursor (CATB), transitional endoplasmic reticulum ATPase isoform 3 (Canis lupus familiaris), heat-shock protein, HSP 90-alpha, fructose-bisphosphate aldolase A (ALDOA), guanine deaminase (GDA), rab GDP dissociation inhibitor beta (GD1B), legumain precursor, metalloproteinase inhibitor 2 precursor (TIMP2), CAP1 protein (CAP1), gelsolin isoform b (GELS), serpin A3-1 precursor (SERPINA31)	Carbonic anhydrase, ezrin, heat shock protein 70, isocitrate dehydrogenase, nucleoside diphosphate kinase, peroxiredoxin 1, purine nucleoside phosphorylase, thioredoxin, triosephosphate isomerise, cystatin, legumain, retinol-binding protein, and tissue inhibitor of matrix metalloproteinase 2.	[32,35,36]
Sheep	Gelsolin isoform b (GSN), Transferrin (TF), Adenosylhomocysteinase (AHCY), Carbonic anhydrase 2 (CA-II), Heat shock 60 kDa protein 1 (HSP60), Apolipoprotein A-1 (APOA1), Galectin 15 (LGALS15/OVGAL11), Ceruloplasmin, Cystatin E/M, Complement C3-like, Cystatin C, Cathepsin L1 isoform 1, Insulin-like growth factor-binding protein-1, Retinol-binding protein 4, Extracellular superoxide dismutase 3, Phosphoglycerate kinase 1, Heat Shock 70kDa Protein 8, Alpha-enolase isoform 1	Transgelin, placental proteins like PP9, component 4 (CC4), immunoglobulin, heavy constant mu (IGHM), adenosylhomocysteinase, glucose 6 phosphate isomerase (GPI), apolipoprotein AI (APO-AI), Ceruloplasmin, A1B glycoprotein (A1BG), actinin 4 (ACTN4), BCL2-like 15 (BCL2-L15), carbonic anhydrase II (CA II), and A2 macroglobulin	[41,42]
Pig	Transferrin, protein DJ-1, transgelin, galectin-1, septin 2, stathmin 1, actin smooth muscle gamma-actin, cofilin 1, fascin 1, heat shock protein (HSP) 90b, HSP 27, serpins, cofilin, annexin A2 (ANXA2), aldose reductase, Annexin A2, cyclophilin, protein disulphide isomerase A3, peroxiredoxin1, haptoglobin, transthyretin, ceruloplasmin Apolipoprotein A-1 (APOA1), F actin capping protein subunit beta (CAPZB), L-Lactate dehydrogenase B chain (LDHB), annexins (ANXA4 and 5), Complement factor B (CFB), B Actin (ACTB), SMS, Actin related protein 3 (ACTR3), Beta enolase (ENO3), Ornithine oxo-acid aminotransferase (OAT), Transthyretin (TTR)	Cathepsin C and B (CTSC, and CTSB), Acid Phosphatase 5 (ACP5)	[44,45,47]

**Table 3:** Key players identified in various studies are essential for embryo implantation during the early phase of pregnancy.

Species	Protein Name	Function	Change in Expression (References)
Bovine	Ezrin	Involved in cytoskeletal rearrangements that facilitate uterine receptivity and embryo-endometrium attachment.	Up (Uterine fluids vs. plasma) [30] Up (Pregnant vs. non-pregnant) [27] Down (Pregnant vs. open) [75]
	Interferon tau	Interferon tau is the signal for maternal recognition of pregnancy in ruminants.	Up (Pregnant vs. open) [75]
	S100-A4	Expressed in the endometrium and play a role in embryo adhesion.	Up (Pregnant with viable embryo vs. Pregnant with degenerate embryo) [62] Up (Day 7 vs. Day 13post estrus) [29]
	Peroxiredoxin-2	PRDX2 is an antioxidant protein that helps in trophoblast proliferation and migration.	Up (Pregnant with viable embryo vs. Pregnant with degenerate embryo) [62] Up (Uterine fluids vs. plasma) [30] Down (Day 15 vs. Day 7) [31]
	Metalloproteinase inhibitor 2 precursor	TIMP-2 is a proteinase inhibitor and functions as a regulator of extracellular matrix integrity by controlling the activity of matrix metalloproteinases.	Up (Day 15 vs. Day 7) [31] Up (Day 16 vs. Days 10,13,19) [32] Down (Pregnant vs. non-pregnant) [27] Up (Day 14 vs. Days 9, 5) [59] Up (Day 7 vs. Day 13post estrus) [29]
	Legumain	LGMN is a lysosomal cysteine protease that plays an important role in implantation and placentation.	Up (Day 15 vs. Day 7) [31] Up (Day 16 vs. Days 10,13,19) [32] Down (Pregnant vs. non-pregnant) [27] Up (Day 14 vs. Days 9, 5) [59] Up (Day 7 vs. Day 13 post estrus) [29]
	Annexin A1 and A2	Annexins are phospholipid binding proteins. Annexin A1 and A2 help in the maintenance of placentation.	Down (normal chorioamnions vs. retarded chorioamnions) [35] Up (Uterine fluids vs. plasma) [30] Down (Day 15 vs. Day 7) [31] Up (Day 9 vs. Days 5) [59]
Sheep	Alpha actinin 4	ACTN4 may act as an important regulator of trophoblast proliferation and differentiation during early pregnancy.	Up (Pregnant vs. Non-pregnant) [41]
	Transgelin	It helps in the formation of podosomes and is involved in tissue invasion and matrix remodeling.	Up (Pregnant vs. Non-pregnant) [41] Up (C vs. IC) [37]
	Integrin, alpha 1	It helps in the attachment of the embryo to endometrial cells.	Up (C vs. IC) [37] Up (IC vs. C areas in the pregnant) [39]
	Annexin	ANXA4 promotes trophoblast cell proliferation and invasion.	Up (implantation (P16) vs. pre-attachment (P12) [38]
	Signal transducer and activator of transcription 1-alpha/beta	Depletion or absence of STAT1 abolishes cell proliferation and invasion of trophoblast cells.	Up (IC of pregnant vs. IC non-pregnant) [39]
	Gelsolin	GSN is involved in the regulation of actin polymerization during early pregnancy.	Down (implantation (P16) vs. oestrous cycle (C16) [38] Up (Non Gravid horn vs. Gravid Horn at day 20) [40]
	Profilin 1	PFN1 is expressed both in the embryo and in the endometrial epithelium and regulates the cytoskeletal organization.	Days 14,16 of pregnancy [43]
Pig	Transgelin,	It helps in the formation of podosomes and is involved in tissue invasion and matrix remodeling.	Up (Non-pregnant vs. pregnant) [44]
	Aldose reductase	ALDR plays an important role in the biosynthesis of prostaglandins and takes part in pregnancy recognition and conceptus development.	Up (day 12 vs. day 9 of pregnant) [45] Up (Pregnant vs. non-pregnant) [46]
	Annexin A2	Annexin A2 helps in the maintenance of placentation.	Up (Pregnant vs. non-pregnant) [46]
	Cyclophilin	CYPA helps in transforming the uterus from a non-receptive state into a receptive state to receive blastocyst.	Up (Pregnant vs. non-pregnant) [46]
	Haptoglobin	HP might contribute to endometrial receptivity for conceptus attachment.	Up (Pregnant vs. non-pregnant) [46] Up (day 12 vs. day 9 of pregnant) [45] Up (9D vs. 16D of pregnancy) [47]
	Annexin A4	ANXA4 promotes trophoblast cell proliferation and invasion.	Up (day 12 vs. day 9 of pregnant) [45] Up (9D vs. 16D of pregnancy) [47]
	Fibrillin-1	These proteins are components of the extracellular matrix and are significantly increased in the endometrium at the time of implantation, suggesting a role in blastocyst attachment.	Up (Day 72 vs. Day 49) [48]

## Data Availability

The data compiled in this review article are collected from the published articles available in PubMed (https://pubmed.ncbi.nlm.nih.gov/) and Google Scholar (https://scholar.google.com/).
